# NSs: the multifaceted bunyavirus virulence factor

**DOI:** 10.1038/s44298-025-00146-5

**Published:** 2025-09-03

**Authors:** Maëva Duboeuf, Anne-Flore Legrand, Pierre-Yves Lozach, Carine Maisse

**Affiliations:** IVPC UMR754, INRAE, Universite Claude Bernard Lyon 1, EPHE, Université PSL, Lyon, France

**Keywords:** Viral host response, Viral immune evasion, Virus-host interactions, Cell death and immune response

## Abstract

*Bunyaviricetes* represents a diverse class of RNA viruses, including hundreds of species distributed worldwide that threaten human and animal health. This Perspective briefly overviews bunyaviruses infecting humans and other mammals, focusing on their key virulence factor, the nonstructural protein NSs. By subverting host cell processes, NSs promotes immune evasion and viral replication. Here, we discuss its role in bunyavirus cytotoxicity and virulence and highlight its implications for pathogenesis.

## Introduction

Until 2017, the *Bunyaviridae* family comprised five genera of arthropod- and rodent-borne viruses with trisegmented, negative-sense RNA genomes related to Bunyamwera virus (BUNV)^1^. In 2017, the International Committee on Taxonomy of Viruses promoted the family into the order *Bunyavirales* and later into the class *Bunyaviricetes*, reflecting the growing diversity of newly identified species^1^. As of 2025, *Bunyaviricetes* includes two orders, more than a dozen viral families, and more than 600 species. Most available information on bunyaviruses comes from studies using a limited number of strains and species. In this *Perspective*, we use the term “bunyaviruses” in a broad sense to refer to all members of the class *Bunyaviricetes*. However, there is a wide variety of bunyavirus species, vectors, hosts, diseases, and geographical distributions. Bunyaviruses are unique in the sense that they infect a broad range of hosts, including humans and other vertebrates, invertebrates, fungi, plants, and protists. Except for arenaviruses and hantaviruses, which are transmitted via contaminated aerosols, most bunyaviruses are arthropod-borne and belong to the supergroup of arboviruses.

The bunyavirus mode of transmission and their ability to cause serious disease in humans and domestic animals, combined with global warming and human activity, highlight the potential of these viruses as agents of emerging disease and their relevance to public and veterinary health. Examples include the *Bandavirus dabieense*, commonly referred to as Dabie virus (DABV) and previously known as severe fever with thrombocytopenia syndrome virus or SFTSV, Toscana virus (TOSV), and La Crosse virus (LACV), which cause thrombocytopenia and hemorrhagic fever, with case fatality rates ranging from 10 to 30%, in Asia; meningoencephalitis in Europe; and pediatric encephalitis in North America, respectively^2–4^. Other examples include Rift Valley fever virus (RVFV) in Africa, Schmallenberg virus (SBV), which emerged in 2011 in Central Europe, and Heartland virus (HRTV) in North America. RVFV infection often results in acute hepatitis, encephalitis, and birth defects in several mammalian hosts, including cattle^5^, SBV is teratogenic in ruminants^6^, and HRTV clusters closely with DABV and shares key virological and biological features^7^. In the absence of vaccines or specific antiviral treatments approved for human use, the World Health Organization has classified two bunyaviruses, RVFV and Crimean-Congo hemorrhagic fever virus (CCHFV), as priority pathogens for which there is an urgent need to accelerate research and development efforts^8^.

We recommend that comprehensive information on the epidemiology, clinical manifestations, and socioeconomic impact of bunyaviruses be obtained from recent studies^9–11^. Here, we provide an overview of the bunyavirus cellular life cycle. We next address the latest insights into how NSs proteins counteract host defenses and responses in humans and other mammals, particularly by modulating innate immunity and cell death to increase viral replication. Finally, we examine the implications for bunyavirus-induced cytotoxicity, virulence, and pathogenesis.

## The bunyavirus cellular life cycle

**Fig. 1:**
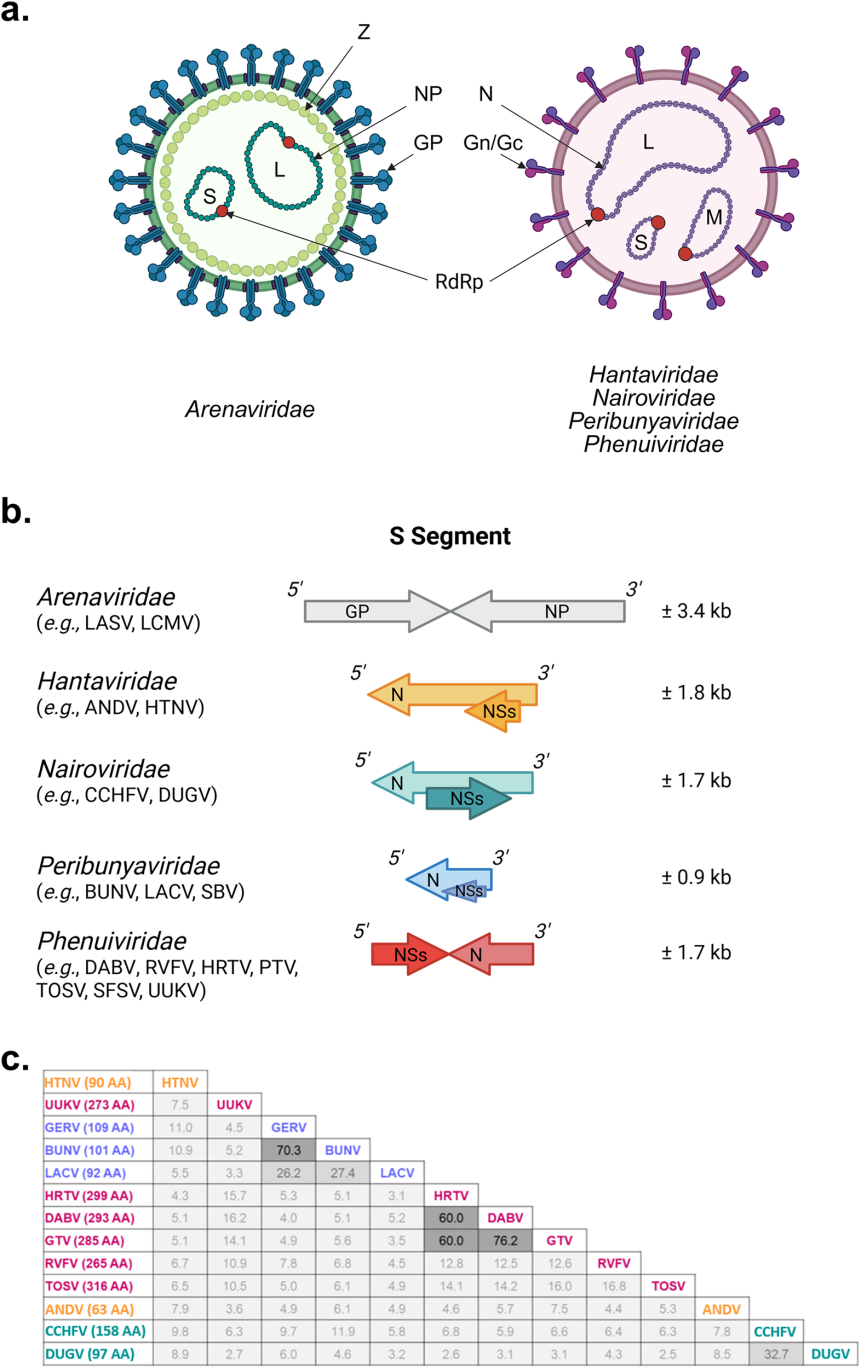
Structural organization of bunyaviruses, their S RNA genomic segment, and their NSs protein. **a** Bunyaviral particles. Schematic representations of typical bunyavirus particles. The viral RNA genome is segmented, and each segment is named according to it relative size: S (small), M (medium), and L (large). **b** Bunyavirus S segment. **c** Percentage identity matrix based on multiple sequence alignment of representative NSs protein amino acid sequences across bunyaviruses. Alignment was performed using MAFFT v7.490. The identity values are color-coded: <25% (light gray), 25–50% (medium gray), and >50% (dark gray). Color code for virus families: *Hantaviridae* (yellow), *Nairoviridae* (green), *Peribunyaviridae* (blue), and *Phenuiviridae* (red). ANDV Andes virus, BUNV Bunyamwera virus, CCHFV Crimean Congo hemorrhagic fever virus, DABV Dabie virus, DUGV Dugbe virus, GERV Germiston virus, Gc glycoprotein c, Gn glycoprotein n, GP glycoprotein, GTV Guertu virus, HRTV Heartland virus, HTNV Hantaan virus, LACV La Crosse virus, LASV Lassa virus, LCMV lymphocytic choriomeningitis virus, N nucleoprotein, NP nucleoprotein, PTV Punta Toro virus, RdRp RNA-dependent RNA polymerase L, RVFV Rift Valley fever virus, SBV Schmallenberg virus, SFSV Sandfly fever Sicilian virus, TOSV Toscana virus, UUKV Uukuniemi virus, Z protein Z. Created in part with BioRender.com.

The diversity within *Bunyaviricetes* is reflected at both cellular and molecular levels. The viral particles are enveloped, roughly spherical, and heterogeneous in size, ranging from 80 to 160 nm in diameter (Fig. 1a). Most bunyaviruses discussed in this *Perspective* have a multisegmented, single-stranded RNA genome, typically composed of three segments of predominantly negative polarity^1^. However, members of *Bunyaviricetes* can display considerable genomic variability, including mono- to polysegmented RNA genomes. The name of a viral genomic RNA segment refers to its size in nucleic acids: “L” for the largest segment, “M” the medium sized segment, and “S” for the smallest segment. Together, they encode four structural proteins, which constitute the viral particles, namely, an RNA-dependent RNA polymerase (RdRp), a nucleoprotein, and two transmembrane glycoproteins. Arenaviruses differ from other bunyaviruses in that they encode a matrix protein (Z), which decorates the inner part of the viral envelope (Fig. 1a). Other bunyaviruses do not have rigid internal structures, and their nucleoproteins therefore play an important role in protecting their genetic information.

**Fig. 2:**
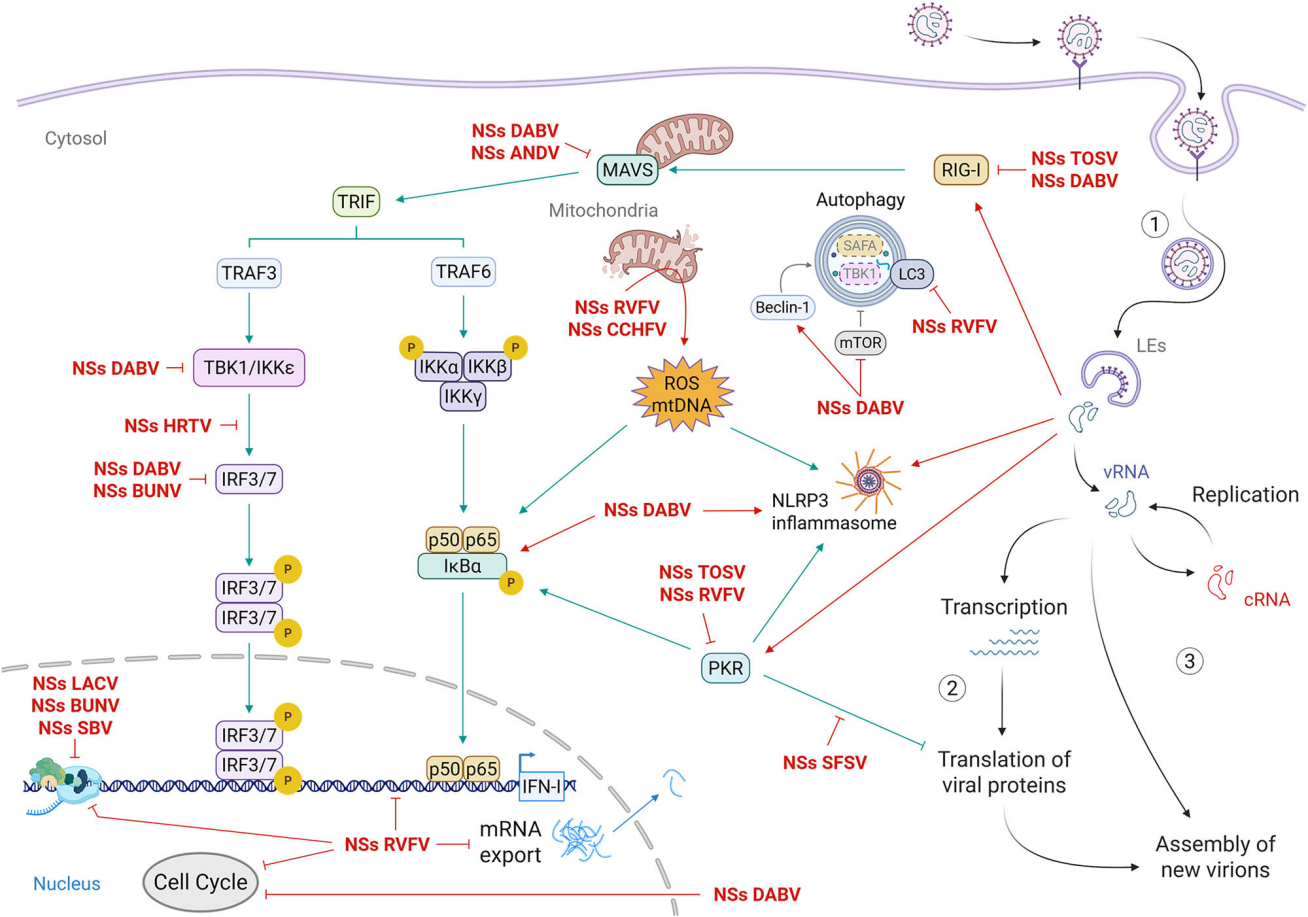
Interaction between the nonstructural protein NSs and the host cellular machinery. Bunyaviruses attach to host cell surfaces via various receptors and are internalized before escaping from late endosomes into the cytosol (1). After genome release, the RNA segments are either transcribed into mRNAs for protein synthesis (2) or replicated for packaging into new virions (3). Throughout the replication cycle, NSs proteins interfere with multiple host antiviral pathways (highlighted in red). ANDV Andes virus, BUNV Bunyamwera virus, CCHFV Crimean Congo hemorrhagic fever virus, cRNA complementary RNA, DABV Dabie virus, HRTV Heartland virus, IFN interferon, IKK IκB kinase, IRF IFN regulatory factor, LACV La Crosse virus, LEs late endosomes, MAVS mitochondrial antiviral-signaling protein, mtDNA mitochondrial DNA, mTOR mammalian target of rapamycin, NFκB nuclear factor kappa B, NLRP3 NOD-like receptor family pyrin domain containing 3, PKR protein kinase R, RIG-I retinoic acid-inducible gene I, ROS reactive oxygen species, RVFV Rift Valley fever virus, SAFA scaffold attachment factor A, SBV Schmallenberg virus, SFSV Sandfly fever Sicilian virus, TBK1 TANK-binding kinase 1, TOSV Toscana virus, TRAF TNF receptor-associated factor, TRIF TIR-domain-containing adapter-inducing IFN-β, vRNA viral genomic RNA. Created in part with BioRender.com.

Each of the viral RNA segments has conserved complementary sequences at the 5’ and 3’ ends that anneal into short double-stranded (ds) structures^12^. The viral RdRp L binds to these terminal sequences, rather than to the annealed structure itself. This assembly results in the pseudocircularization of each segment, which typically has a panhandle or hairpin appearance within the viral particles^12^. Within a viral particle, the nucleoprotein is associated with the RNA genome and, together with the viral RdRp, forms ribonucleoproteins (RNPs)^12^. On the surface of each viral particle, the transmembrane glycoproteins constitute protrusions responsible for the attachment of the virus to a host cell^13^. Bunyaviruses enter target cells via receptor-mediated endocytosis, followed by fusion of the viral envelope with endosomal membranes under low pH conditions and the release of viral RNPs into the cytosol (Fig. 2)^14–16^. Viral replication begins, and the cell becomes infected.

Bunyaviral replication, as well as the assembly and release of infectious progeny particles, involve complex mechanisms that remain largely uncharacterized. However, the viral genomic RNA segments replicate exclusively in the cytosol of infected cells. The negative polarity of the viral genome prevents the translation of viral RNA segments by the host cell machinery. Instead, viral messenger RNAs (mRNAs) are transcribed, exhibiting a 5’-cap and a short stretch of a heterogeneous sequence, both of which are derived from the host cell, but no poly(A) tail^17^. The nucleoprotein interacts with viral genomic RNAs as they are being synthesized and favors their incorporation into nascent viral particles. Another exceptional trait of arenaviruses is that they leave cells via the plasma membrane^18^. In contrast, other bunyaviruses assemble and bud in the Golgi network, from which the viral particles acquire their lipidic bilayer envelopes before exiting host cells^15,19^. The viral progeny ensures the transfer of the viral genomes from infected cells to uninfected cells, although other mechanisms, notably cell-to-cell transfer, have been proposed for bunyaviruses^20,21^. Excellent reviews have addressed the most recent knowledge regarding the molecular mechanisms of bunyavirus replication^12,17,22^.

## Bunyavirus nonstructural proteins

Except for arenaviruses, bunyaviruses encode up to two nonstructural (NS) proteins, NSm and NSs. The number of NS proteins varies across viral families, genera, and even strains, and their expression has yet to be confirmed in many cases. However, most bunyaviruses encode an NSs protein (Fig. 1b), although the corresponding sequences are highly divergent across species, as illustrated in Fig. 1c. The NS proteins are typically small (10–40 kDa) and expressed in infected cells, but not incorporated into viral particles^23–25^. In the case of arenaviruses, the nucleoprotein N and the matrix protein Z, two structural proteins, appear to affect the functions of the NS proteins of other bunyaviruses. The specific case of arenaviruses will only be partially addressed, as we will be concentrating on NS proteins here. We recommend the excellent review by Stott and colleagues for more details regarding arenaviruses and the N and Z proteins^18^.

The bunyavirus NS proteins are generally not required for viral replication, but they play an essential role in virus amplification and propagation. They efficiently modulate host cell functions and defenses. NSm has been shown to facilitate the spread of RVFV by inhibiting the apoptosis triggered by infection^26^. In insect cells, an alternative form of NSm is thought to play a structural role in virions^27^ and appears to be a key determinant in bunyavirus transmission between mosquitoes and vertebrates^28^. However, the NSm protein remains largely uncharacterized beyond these findings. The NSs protein is somewhat better studied, although most of the available data pertain to those of RVFV, and to a lesser extent, DABV.

## The virulence factor NSs, a versatile viral protein

The accumulation of evidence has indicated that the bunyavirus NSs protein is a major virulence factor, with a significant contribution to disease outcomes in mammalian hosts. A notable example is RVFV. A natural RVFV strain lacking most of the NSs sequence, clone 13, propagates in BALB/c mice; however, the animals are able to control viral replication, clear the infection, and survive^29^. In contrast, infection with the wildtype (WT) RVFV strain induces nonlethal acute hepatitis, followed by fatal encephalitis, mirroring what is seen in severe human cases^29^. Similar observations have been reported regarding BUNV, DABV, HRTV, SBV, and Punta Toro (PTV)^30–36^. Genetically engineered deletion of the NSs gene in the S segment attenuates each virus. Collectively, these studies establish NSs as the principal virulence factor of mammalian bunyaviruses.

Several observations have supported the hypothesis that NSs functions are correlated with bunyavirus dissemination and disease severity. Bunyavirus NSs proteins exhibit diverse biological activities and generally act as multifunctional regulators of molecular and cellular processes. NSs has been shown to play a crucial role in the host antiviral defense evasion ability of several bunyaviruses. Therefore, NSs likely facilitates virus amplification and propagation throughout the host, although NSs has not been reported to be directly involved in viral replication and progeny production^37,38^. The following sections explore the various biological functions of NSs that contribute to molecular and cellular disorders, with a potential link to promote viral replication, cause cytotoxicity, and increase disease severity.

## NSs, the host cellular machinery, and viral replication

Several observations have indicated that NSs proteins encoded by species from different viral families cause a general shutdown of host gene expression following infection. First, RVFV NSs interacts with the p44 and p62 subunits of the TFIIH transcription complex, thereby inhibiting overall host transcription^39^. Similarly, the BUNV, LACV, and SBV NSs proteins suppress host gene expression by targeting RNA polymerase II (RNA Pol II), which is essential for cellular mRNA synthesis^40–44^. BUNV NSs was first shown to prevent phosphorylation of the C-terminal domain of the RNA Pol II and impair the transition of initiation-to-elongation RNA synthesis^43^. This process was later proposed to involve the interaction of NSs with MED8^40^, a subunit of the RNA Pol II preinitiation complex. In contrast, LACV and SBV NSs promote the proteasomal degradation of the RPB1 subunit of RNA Pol II^41,44^; this process has been shown to involve the E3 ubiquitin ligase Elongin C and its cofactors Elongin A and B in the case of LACV^41,42^. In addition, RVFV NSs interacts with the nuclear pore and disrupt nuclear transport, resulting in the accumulation of host mRNAs in the nucleus^45–47^. In contrast, the NSs protein of the bunyavirus Uukuniemi virus (UUKV) does not appear to affect cellular transcription or translation^48,49^. Unlike the NSs proteins of RVFV, LACV, SBV, and BUNV, which translocate to the nucleus^41,50–52^, UUKV NSs remains strictly cytoplasmic, suggesting a correlation between NSs nuclear translocation and the inhibition of general transcription. Overall, NSs undoubtedly makes it easier for bunyaviruses to subvert host cellular machinery for their own purposes and replicate their genetic materials.

## Antiviral responses: the interplay between NSs and cellular sensors

The host immune system employs two main lines of defense against invading pathogens: innate and adaptive immunity. While adaptive immunity against bunyaviruses remains poorly documented, the production of neutralizing antibodies, which primarily target viral glycoproteins on the surfaces of viral particles, appears to be critical for controlling infection across mammalian hosts^25^. For the vast majority of bunyaviruses, dermal dendritic cells and macrophages are among the first cells to encounter incoming viral particles, and each cell type is highly susceptible to infection^53^. Additionally, the rapid proliferation of neutrophils, B-cells, and CD4^+^ and CD8^+^ T-cells, which are key players in antiviral immunity, seems to be correlated with a reduced severity of disease in both humans and primates exposed to RVFV^25^.

Innate immunity is also crucial for limiting bunyavirus pathogenesis, and the NSs proteins of several bunyaviruses are well-known antagonists of this response^38,54–56^. Briefly, innate immune activation relies on numerous cellular sensors, collectively known as pattern recognition receptors (PRRs), that detect various molecular components of pathogens, such as viral nucleic acids and proteins. PRR activation typically leads to interferon (IFN) induction and the release of IFNs and proinflammatory cytokines. For example, PRRs such as RIG-I-like receptors, certain Toll-like receptors, and protein kinase R (PKR) specialize in the sensing of viral RNA. This triggers a kinase signaling cascade that leads to the phosphorylation and nuclear translocation of NFκB (p65) and IFN regulatory factor 3 (IRF3), ultimately resulting in the production of type I IFN (IFN-I). For a comprehensive review of these key players in innate immune activation, we refer readers to the recent review by Dalskov et al.^57^.

There is a growing body of evidence that bunyaviruses have evolved strategies to target PRRs and suppress antiviral defenses via NSs^38,55,58^. Among these strategies, the NSs protein of RVFV is the most extensively studied and is known to be a potent silencer of PKR^59,60^. A hallmark of RVFV infection is the formation of striking nuclear filaments, which were first reported by Swanepoel and colleagues in 1977^50^. These structures have recently been identified as bundles of thin (10-nm wide) NSs fibrils resembling amyloid-like aggregates^61^. Recent structure–function studies have indicated that these fibrillar aggregates play a key role in PKR silencing and other biological functions of NSs^59,61^. Mechanistically, RVFV NSs recruits an E3 ubiquitin ligase complex comprising Skp1, Cul1, FBXW11, and β-TRCP1 to promote PKR degradation via the proteasome, thereby preventing the amplification of IFN-I responses^62–65^. This also counteracts the PKR-mediated translation shutdown, allowing continued protein synthesis in infected cells. Similarly, the NSs protein of TOSV has been proposed to interact with PKR and promote its proteasomal degradation, although the specific molecules involved remain to be identified^66^ (Fig. 2). In contrast, studies on Sandfly fever Sicilian virus, a bunyavirus closely related to TOSV, have shown that NSs does not induce PKR degradation^67^ but instead sustains viral protein synthesis by binding to the translation initiation factor eIF2B and preventing its inhibition by PKR-phosphorylated eIF2α^68^, thereby bypassing the effects of PKR activation (Fig. 2).

Other bunyaviruses adopt distinct strategies to disrupt PRR signaling. For example, DABV NSs forms cytoplasmic inclusion bodies (IBs), large structures in which NSs accumulates and that sequester PRRs and associated host factors such as TRIM25, an E3 ubiquitin ligase essential for RIG-I ubiquitination and activation. By trapping TRIM25, DABV NSs prevents the sensor activity of RIG-I and thereby blocks IFN production at an early stage of PRR signaling. Ectopic expression of TOSV NSs has been shown to suppress IFN responses by promoting RIG-I degradation, potentially through an intrinsic E3 ubiquitin ligase activity^69^ (Fig. 2). In addition, the NSs protein of Andes virus, a highly pathogenic hantavirus, antagonizes the innate immune response by interfering with IFN signaling downstream of RIG-I and upstream of TANK-binding kinase 1 (TBK1), likely through disrupting MAVS signaling^70^. The precise mechanism, however, remains to be fully elucidated.

Similarly, arenaviruses block and evade the IFN response through their Z proteins. Specifically, Z prevents the interaction of RIG-I and the other dsRNA sensor, melanoma differentiation-associated protein 5 (MDA5), with mitochondrial antiviral-signaling protein (MAVS), which is essential for activating IRF3, IRF7, and p65 (Fig. 2). Furthermore, arenaviruses utilize their nucleoproteins to degrade dsRNA replication intermediates, thereby circumventing PRR activation^18^. Through these mechanisms, arenaviruses manage to finely regulate viral genomic RNA replication to sustain progeny production while evading the innate immune system.

## Autophagy and antiviral responses

Recent studies have implicated autophagy in the host antiviral response to both DABV and RVFV (Fig. 2). DABV NSs colocalizes with the autophagy-related proteins LC3B and p62^71^, and promote autophagy induction by inactivating mTOR, a repressor of autophagy^72^. Consistently, NSs has been shown to activate Beclin1, a key positive regulator of autophagy, leading to enhanced autophagic activity and increased DABV replication^72,73^. NSs-induced autophagy has also been associated with the degradation of TBK1 and the RNA sensor scaffold attachment factor A (SAFA)^74,75^. While SAFA degradation requires NSs-mediated interactions with LC3^75^, it remains unclear whether Beclin1 activation results from a direct interaction with NSs^73,74^, and whether sequestration within NSs-induced IBs or canonical autophagic degradation is involved.

Collectively, these findings support a model in which NSs-triggered autophagy facilitates immune evasion and favors DABV replication. In contrast, RVFV NSs contains multiple LC3-interacting regions and have been proposed to suppress autophagy in infected cells^76^. However, whether autophagy plays a pro- or antiviral role during RVFV infection remains debatable^77,78^. Together, these observations highlight the complex and virus-specific interplay between bunyavirus NSs proteins and the autophagy machinery, and whether it promotes viral replication or contributes to antiviral defense remains to be investigated for most bunyaviruses.

## NSs, a potent antagonist of IFN induction and response

The NSs proteins of several hantaviruses and other pathogenic bunyaviruses interfere with the IFN pathway downstream of PRR signaling inhibition^24,38,55,58,79–83^. In RVFV-infected cells, nuclear NSs filaments retain the transcription factors Sin3A-associated protein (SAP30) and YY1. SAP30 is a transcriptional repressor that plays a central role in regulating gene expression and maintaining chromatin stability through histone deacetylation. Together with YY1, SAP30 forms a molecular complex that prevents the transcriptional coactivator CREB-binding protein (CBP), an acetyl transferase, from accessing the IFN-β promoter, thereby repressing IFN-β gene expression^84^. Additionally, this interaction leads to chromosome segregation defects during cell division^84^. In parallel, SBV NSs has also been reported to localize to the nucleolus, where it induces nucleolar disorganization^52^. A potential link has been proposed between this disruption and the ability of NSs to suppress host transcription, thereby precluding cellular antiviral responses^52^.

Similarly, the NSs proteins of DABV and HRTV inhibit IFN-β responses at steps subsequent to PRR activation. First, the cytosolic IBs formed by DABV NSs sequester the downstream signaling proteins IκB kinase ε (IKKε) and TBK1, which are responsible for phosphorylating IRF3 and IRF7^58,85,86^. As a result, the transcription factors IRF3 and IRF7 remain unphosphorylated, which prevents their translocation into the nucleus (Fig. 2). HRTV NSs similarly inhibits IRF3 activation by preventing its association with TBK1^87^. In addition, DABV NSs has been shown to also sequester IRF7^88^. Altogether, these interactions lead to a general suppression of IFN gene expression and IFN production.

Both DABV and HRTV NSs disrupt IFN signaling at a later stage by interfering with receptor-mediated responses^89–92^. Similar to its effect on MAVS, TBK1, and IKKε, DABV NSs captures the transcription factors STAT1 and STAT2 in IBs. Consequently, these factors are no longer phosphorylated upon IFN receptor activation, effectively abolishing the IFN response. In contrast, HRTV NSs selectively impairs the nuclear translocation of STAT2 without affecting STAT1^91,92^, suggesting a more targeted mechanism. However, the reduced phosphorylation of both STAT1 and STAT2 in HRTV-infected cells, along with the overall suppression of IFN signaling, suggests that additional viral factors beyond NSs are likely involved.

Regarding arenaviruses, the nucleoprotein NP and the matrix protein Z appear to compensate for the absence of NSs by fulfilling similar functions. For example, NP has been shown to suppress the IFN response by inhibiting key components of the RIG-I/MDA5 signaling pathway, including IKKε and IRF3^93–95^.

## Inflammatory responses, cell death, and fatal host defenses

In addition to limiting viral replication in infected cells, the IFN response alerts neighboring, uninfected cells to the presence of a potentially harmful pathogen. However, cells that emit such a danger signal in response to infection often undergo regulated cell death, either via an inflammatory mode, such as pyroptosis, or via a noninflammatory mode, primarily apoptosis. Pyroptosis is initiated in the cytosol by activation of the inflammasome in response to cellular stress or danger signals. This multiprotein platform ultimately activates caspase-1, a key enzyme that promotes pore formation in the plasma membrane and the release of proinflammatory cytokines, thereby recruiting immune cells to the site of infection^96^. In contrast, apoptosis, a “silent” form of cell death, can be mediated either by p65-induced and IRF3-induced activation of death receptors at the plasma membrane or by mitochondrial damage.

In this context, the inflammatory response and regulated cell death represent ultimate host defense strategies, limiting viral spread by restricting the exposure of adjacent cells to the invading pathogen. The contribution of cellular stress responses, particularly cell death, to host antiviral defense mechanisms, both at the cellular scale and at the whole-organism level, is often underestimated. Importantly, cell death acts as a double-edged sword during viral infection. While it facilitates pathogen clearance and promotes the recruitment of immune cells to initiate adaptive immunity, excessive or highly immunogenic cell death can exacerbate pathogenicity at the systemic level. Like many other viruses, bunyaviruses commonly induce cytotoxicity and inflammation. Notably, the NSs protein has been implicated in these processes, suggesting that NSs may be a key determinant of the pathophysiological outcomes associated with bunyavirus infection.

Specifically, RVFV NSs expression impedes the actin cytoskeleton by inhibiting Abl2, a critical regulator of the actin network, leading to pronounced morphological alterations^97^. Furthermore, RVFV NSs and DABV NSs induce cell cycle arrest through distinct mechanisms (Fig. 2). In RVFV-infected cells, NSs promotes the phosphorylation of p53^98,99^, a posttranslational modification that plays a key role in halting cell cycle progression. Conversely, DABV NSs sequesters CDK1 within IBs and prevents the formation of the cyclin B1-CDK1 complex, which is also essential for cell cycle progression^100^. In both cases, the resulting cell cycle arrest enhances viral replication.

Severe DABV infection is marked by a cytokine storm^101,102^ driven by excessive activation of p65^103^ and likely also by NLRP3^104–106^, a pivotal innate immune sensor within the inflammasome^96^. The NSs protein undoubtedly contributes to these exacerbated inflammatory processes. It has been shown to increase p65 activation^103^ and to directly interact with NLRP3, promoting caspase-1 activation and, in turn, pyroptosis and inflammation^105^. Conversely, it is noteworthy that DABV NSs appears to induce the production of the immunosuppressive cytokine IL-10 by targeting the TPL2 signaling pathway^107^ and, more specifically in macrophages, by promoting the overexpression of miR-146b^108^, thereby dampening host defenses. This dual ability of NSs to both activate NLRP3 and promote IL-10 raises intriguing questions about how DABV fine-tunes host immune responses to support viral persistence and pathogenesis.

Overexpression of the PTV NSs protein has been shown to induce caspase activation, leading to apoptosis^109^. CCHFV NSs has been found to disrupt mitochondrial membrane potential via its C-terminal region (Fig. 2), leading to caspase activation and apoptosis^110^, although its precise role in regulating apoptosis during infection remains to be fully defined. In RVFV-infected cells, NSs colocalization with mitochondria correlated with elevated levels of reactive oxygen species, which also led to the activation of the p65 and p53 signaling pathways (Fig. 2)^111^. This finding is consistent with a potential contribution to mitochondrial dysfunction and loss of integrity, although a direct causal effect of NSs has not yet been established. Furthermore, RVFV NSs induces mitochondrial damage through the transcriptional downregulation of the antiapoptotic protein MCL1^112^. This results in the release of mitochondrial DNA, which can further activate the inflammasome via NLRP3^112^, as has also been observed regarding DABV^106^.

Conversely, alternative strategies involving the use of NSs proteins to modulate the balance between cell survival and death have been developed by some bunyaviruses. For example, activated caspase-3 accumulates within nuclear NSs filaments in RVFV-infected cells, preventing apoptosis^113^. In addition, BUNV NSs appears to counteract the activity of the transcription factor IRF3 and delay apoptosis (Fig. 2)^114^. While IRF3 is a crucial regulator of the innate immune response, it also performs proapoptotic functions, not only through its transcriptional activity but also via posttranslational interactions^115^. These findings suggest that IRF3 is a key link between antiviral immunity and regulated cell death.

Collectively, these reports suggest that while NSs proteins play a common role in targeting innate immune responses, their impact on cell death processes varies significantly among bunyaviruses. In any case, current evidence strongly supports the notion that NSs proteins are key drivers of the cytotoxicity associated with bunyavirus pathogenesis. It remains unclear whether NSs-mediated modulation of cell death is a primary function of the protein or a secondary consequence of its other activities. Nonetheless, while NSs may ultimately trigger cell death, the virus appears to have sufficient time to complete its replication cycle. Bunyaviruses typically complete their replication within 5–6 h, whereas infected cells generally undergo apoptosis no earlier than 24 h post-infection.

## Future perspective

Bunyaviruses exhibit remarkable diversity at both the molecular and cellular levels, with significant variations in genomic organization and structural features across families. This diversity is particularly evident in the encoding strategy, size, and sequence identity of NSs, the least conserved bunyavirus protein, whose sequence identity ranges from 8 to 29% (Fig. 1c)^116^. This raises the legitimate question of whether all these highly divergent proteins should retain the same “NSs” designation, which is currently based primarily on their genomic location within the S segment, rather than on sequence similarity. To date, half a dozen NSs proteins have been analyzed, most of which originate from pathogenic bunyaviruses and share the ability to counteract innate immune defenses. Further studies using a broader range of bunyavirus strains and species are necessary to determine whether, despite their apparent molecular diversity, all NSs proteins serve the same biological function in mammalian hosts, mainly facilitating viral immune evasion.

Accumulating evidence suggests that NSs proteins also disrupt cellular viability. This dual role likely underpins their function as virulence factors. By interfering with immune responses and cell death pathways, NSs may influence disease severity. For example, the NSs protein of UUKV, which is not associated with any mammalian disease, exhibits only weak IFN antagonism^48^, unlike the NSs proteins of the highly pathogenic bunyaviruses RVFV and DABV. Further investigations are essential to delineate the connections between NSs, immunity, cell death, and disease severity. Evidently, NSs has emerged as a valuable tool for exploring hard-to-investigate molecular mechanisms involved in the interplay between innate immunity and cell death pathways, a field that remains largely unexplored.

NSs filaments are a hallmark of RVFV infection, yet similar structures have not been reported for other bunyaviruses. However, this does not preclude their existence. First, tools for studying the structure and cell biology of all NSs proteins are lacking. In addition, the NSs proteins of SBV and DABV do not form filaments under fluorescence microscopy observation but instead form large amorphous aggregates in the nucleus and cytoplasm, respectively^52,86^. Such structures have also been reported for RVFV NSs^61^. Electron micrographs clearly show that they consist of an accumulation of very thin, short fibrils^61^. It is therefore tempting to postulate that NSs also assembles into fibrils in cells infected with SBV and DABV, and also with other bunyaviruses. The biological functions and roles of such NSs aggregates in infection, if any, would remain to be elucidated.

Viral protein aggregates have also been reported regarding unrelated viruses. These aggregates often exhibit phase-separating properties, which are well known to facilitate the segregation of viral genetic material and the formation of specialized microenvironments that promote viral replication and the assembly of new viral particles^117^. NSs has not been associated with such functions; however, a growing body of evidence highlights its influence on the dynamics and extent of amplification and spread of several bunyaviruses^118^. It is also tempting to draw a link between the aggregation of certain NSs proteins and the formation of IBs observed during infection with some bunyaviruses.

While IFN antagonism is undoubtedly a contributing factor, NSs also modulates host cell machinery, creating a favorable environment for viral replication and dissemination. Investigating these aspects remains challenging, not because reverse genetics approaches are inherently flawed, but because genetic manipulations of the NSs-coding region can introduce confounding effects. In many cases, introducing point mutations or tags reduces NSs expression, making it difficult to distinguish a true loss of function from simply lower protein levels. In addition, such modifications can alter the organization and structure of the viral genomic RNA, thereby influencing the expression of other viral proteins rather than solely targeting NSs. Although reverse genetics has been highly informative in studying NSs functions, these limitations call for cautious interpretation and the use of complementary approaches.

The S-genomic segment of RVFV is relatively malleable, and strains lacking NSs replicates in peripheral tissues following transmission to mammalian hosts. Unlike the WT virus, these strains do not reach secondary target organs such as the liver and brain, underscoring the critical role of NSs in RVFV dissemination. The NSs protein indirectly shapes viral tropism and represents a promising model for studying the complex biology of zoonotic virus spread. In contrast to most other zoonotic viruses, which often encode multiple NS proteins, many bunyaviruses rely heavily on NSs to support replication and evade host immune responses. Most bunyaviruses of interest also encode an NSm protein, the other NS protein that can influence both vertebrate-to-arthropod transmission and replication in arthropod vectors, as notably observed for BUNV^28^ and RVFV^27,119,120^. Nevertheless, the central role of NSs is particularly striking in the life cycle of viruses such as DABV and HRTV, which lack NSm altogether.

The transition from vector to host implies a profound biological shift, particularly when moving from arthropods to mammals, and influences molecular components and cellular processes. Unlike the cytotoxic effects observed in mammalian cells, infections by bunyaviruses and other arboviruses in arthropods are typically asymptomatic and persistent^121,122^. Interestingly, bunyavirus persistence in mosquito cells seems to rely on the downregulation of NSs expression by a specific RNA interference response^123,124^. Overall, the role of NSs in arthropods remains poorly understood, and their potential involvement in the vector-to-host transition is entirely unknown. Expanding research to a wider range of bunyaviruses and incorporating NSm are essential for elucidating NSs functions in arthropod viral persistence, species barrier crossing, and often fatal outcomes of infection in mammals. Unraveling these mechanisms is crucial for addressing why mammalian hosts often succumb to infection while arthropods survive.

## Data Availability

No datasets were generated or analysed during the current study.
